# Annotations

**Published:** 1896-08-08

**Authors:** 


### ANNOTATIONS.

### Milk Typhoid in Scotland.

The history of an outbreak of typhoid fever in the village of Fintry, published in his last annual report by Dr. John C. McVail, M.O.H. for Stirlingshire, furnishes an object-lesson to sanitarians which will well repay study. The village itself consists of a single straggling street, and the total population at the period of the outbreak numbered 150 persons. Of these no fewer than twenty-two, or one in seven, were attacked by typhoid fever, and all, so far as the most careful inquiries which could be made could determine, as the result of drinking milk supplied by one small dairy of not more than two or three cows. The details of the investigation we have not space for. But the time and trouble given to the discovering of the cause of the outbreak were enormous, and left practically no doubt that this one small dairy in a very few weeks produced a condition of things which, if it could be paralleled in London, would give us an epidemic of typhoid attacking more than half-a-million persons. It is impossible to exaggerate the sanitary significance of this village outbreak, the more especially that, in Dr. McVail's judgment, it was entirely preventable, and would have been prevented but for the foolish condition of the law relating to dairies. Ordinary dairies are, of course, subjected to constant inspection, and the dangers of epidemics of fever from them are reduced to a minimum. But small dairies of two or three cows are specially exempted from inspection; with such results as the disgraceful outbreak at Fintry. Dr. McVail extended his investigations widely, and found many small dairies, not inspected, in a shockingly insanitary condition; and we know of our own knowledge that in England as well as in Scotland, and probably still more in Ireland and Wales, the small dairyman is about as ignorant and stupid a person in matters sanitary as can well be found. As Dr. McVail insists, all dairies, and we would add, all food-producing agencies of every kind, should be forthwith placed under competent sanitary inspection.

### The Holidays of the Married.

No person who has been married for a longer period than six months will doubt for a moment that husband and wife are happier for being sometimes parted. Even Mr. G. R. Sims, who, we believe, is a fully-fledged bachelor, knows this by instinct and the light of nature. We are not surprised, therefore, to find him writing with unhesitating authority on the subject of "Husbands and Holidays." Mr. Sims advocates that the husband—every husband—should spend his holiday apart from his wife, every wife at a distance from her husband, and their children in some place where they are not under the direct supervision of their parents. The theory has its attractions. For our part, we have to look at it from the physiological and hygienic standpoints. "Friction" is the idea that presents itself to the medical mind when it thinks of the associated wife and husband; and when half-a-dozen children are brought into the field of vision, "friction" looms larger and larger still. If it be said that there ought not to be any friction between properly constituted married couples, the answer of medical experience is that there is friction; that it is more or less universal; and that sometimes, nay frequently, it is so very considerable as to be decidedly injurious to health. If this be so, and it is, then there are really but two questions to be asked and answered in order to a medical decision. And first, is prolonged nervous friction a bad thing for the brain and body? To which physiology answers "Yes." And, secondly, is relief from friction for as long a period as possible a distinct advantage to brain and body? And again the answer must be "Yes." So far then as medicine is concerned Mr. George R. Sims, bachelor, must be held to have pronounced with wisdom on this highly interesting marital question. But there are, of course, other aspects of the case, notably the economic one. If holidays are to mean three establishments instead of one then the grim spectre of a worse kind of "friction" namely, the friction resulting from deficient "wherewithal" may be evoked; and so the cure may be worse than the disease. On the whole then, whilst separate holidays may be a counsel of perfection which those may adopt who can, it will probably be necessary for the average husband and wife, with their children, to make themselves as happy as possible together in their seaside nest just as they do at home.

### Is Consumption on the Decrease?

Whilst there are some grounds for fearing, notwithstanding the explanation of "improved diagnosis," that cancer may be on the increase, there seems to be no doubt at all that phthisis is decreasing in Great Britain. A period of 42 years, with careful calculations of statistics, is long enough to justify a generalisation; and that is the period which Dr. Tatham, following in the footsteps of Dr. Farr and Dr. Ogle in the publication of a "Decennial Supplement," has brought under review. From 1853 to 1895 there has been a continuous decrease in the mortality from phthisis of a very marked kind. Of course, this also may be partly accounted for by "improved diagnosis": for, as is well known, whenever the ordinary practitioner of the old school was in doubt about a diagnosis for a case of wasting disease, he wrote down "phthisis" without a moment's hesitation. But this notwithstanding, it seems certain that phthisis is less prevalent among us than formerly; and common experience is in accord with Dr. Tatham's figures. The facts are sufficiently striking. Whereas in 1853 there was a phthisis mortality of 2,984 per million, the mortality in 1890 had fallen to 1,082; and this rate of decrease has been continuously maintained up to the present time. Is this due to improved methods of treatment, or to improved general sanitation? We incline to think it is due to both. There is no doubt that incipient phthisis is much more decidedly recognised now than formerly, and that climatic and other treatment is much more prompt and thorough. But it seems equally clear that in consequence of improved general sanitation, and especially of the housing of the poor in better homes, and drier and more wholesome sites, the prevalence of phthisis is greatly diminished throughout the community. All this may be claimed as a triumph for scientific medicine. And, as we so often insist here, the doctor, in labouring so hard to prevent disease, is "killing the goose which lays the golden eggs." But what thanks or recognition does he receive at the hands either of the public of the State?

